# Coronary artery calcium scoring: expanding the new standard by photon-counting detector CT—Part I: Impact of tube voltage, tube current, slice thickness, and quantum iterative reconstructions

**DOI:** 10.1007/s00330-026-12355-4

**Published:** 2026-02-19

**Authors:** Nicola Fink, Lennart R. Koetzier, Emese Zsarnoczay, Milan Vecsey-Nagy, Dmitrij Kravchenko, Muhammad Taha Hagar, Jim O’Doherty, Moritz C. Halfmann, Pal Suranyi, Gijs D. van Praagh, Jens Ricke, Pal Maurovich-Horvat, Tobias Bäuerle, Martin J. Willemink, Akos Varga-Szemes, Tilman Emrich

**Affiliations:** 1 Department of Radiology and Radiological Science, Medical University of South Carolina, Charleston, SC USA; 2 Department of Radiology, University Hospital, LMU Munich, Munich, Germany; 3 Department of Radiology, Stanford University School of Medicine, Stanford, CA USA; 4 Department of Radiology, Medical Imaging Centre, Semmelweis University, Budapest, Hungary; 5 Heart and Vascular Center, Semmelweis University, Budapest, Hungary; 6 Department of Diagnostic and Interventional Radiology, University Hospital Bonn, Bonn, Germany; 7 Department of Diagnostic and Interventional Radiology, Medical Center–University of Freiburg, Faculty of Medicine, University of Freiburg, Freiburg, Germany; 8 Siemens Medical Solutions, Malvern, PA USA; 9 Department of Diagnostic and Interventional Radiology, University Medical Center of Johannes Gutenberg-University, Mainz, Germany; 10 German Centre for Cardiovascular Research, Partner site Rhine-Main, Mainz, Germany; 11 MTA-SE Cardiovascular Imaging Research Group, Department of Radiology, Medical Imaging Centre, Semmelweis University, Budapest, Hungary

**Keywords:** Coronary artery calcium, Photon-counting detector CT, Agatston score, Phantom study, CT protocol

## Abstract

**Objectives:**

Coronary artery calcium (CAC) scoring is a well-established method for cardiovascular risk assessment but has limited reproducibility. For energy-integrating detector (EID)-CT, a new, multivendor validated protocol has been proposed. This study aimed to investigate the variability of photon-counting detector (PCD)-CT-based CAC scoring and propose a new protocol with decreased variability.

**Materials and methods:**

A chest phantom containing nine calcifications was scanned on a PCD-CT using various settings: tube voltages (90 kVp, 120 kVp), tube currents (100% to 25% dose), slice thickness (3 mm, 1 mm), quantum iterative reconstruction (IR, 1–4). To evaluate interscan variability, phantoms were scanned five times per protocol with slight translational (5 mm) and rotational (2°) movements. The standard PCD-CT protocol used 120 kVp, 100% dose, 3 mm slices. CAC scores, image noise, and calcification detectability were assessed. Results were compared to the standard PCD-CT, and standard and proposed EID-CT protocols.

**Results:**

Compared to the standard PCD-CT protocol, score variability decreased by 37% using a thin-sliced protocol at 120 kVp, 25% dose reduction and IR2. Compared to the proposed EID-CT protocol, variability was 66% lower. The optimized PCD-CT protocol met noise targets, eliminating the risk of false-positives. While 6.0 ± 0.0 and 7.0 ± 0.4 calcifications were detected using the PCD-CT standard and the proposed EID-CT protocol, respectively, 7.1 ± 0.7 calcifications were detected with the optimized PCD-CT protocol. Volume and mass scores were closer to physical reference.

**Conclusions:**

A thin-slice, 25%-dose-reduced PCD-CT protocol at 120 kVp improves CAC score reproducibility and outperforms the proposed EID-CT protocol, possibly offering more reproducible CAC quantification at lower radiation doses.

**Key Points:**

***Question*** *Coronary artery calcium scoring is used for cardiovascular risk stratification. However, the current standard method lacks score reproducibility.*

***Findings*** *A thin-slice, 25%-dose-reduced photon-counting detector CT protocol at 120 kVp significantly reduces score variability compared to previous protocols, including the proposed energy-integrating detector CT protocol.*

***Clinical relevance*** *Improved reproducibility of coronary artery calcium scoring may enable more consistent cardiovascular risk prediction and provide a robust technical basis for further in vivo studies.*

**Graphical Abstract:**

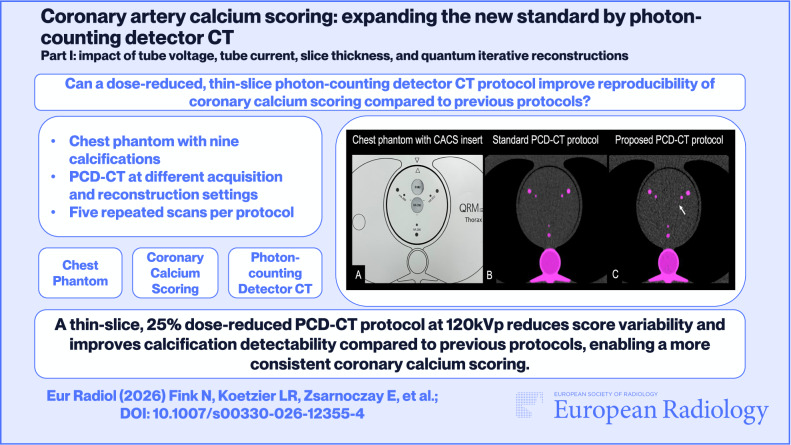

## Introduction

Coronary artery calcium (CAC) scoring is known as a strong predictor of adverse cardiovascular events [1–3]. The 2019 American College of Cardiology/American Heart Association Guideline on the Primary Prevention of Cardiovascular Disease recommended CAC scoring for reclassification of borderline or intermediate risk patients to improve clinical decision-making [4]. Although CT technology has improved significantly in the last decades, CAC quantification is still based on the recommendations given by McCollough et al in 2007 [5] using 120 kVp and 3 mm slices, which underlines the need for a new standard in CT-based CAC scoring [6]. This is further supported by evidence showing that CAC score reproducibility using the current standard method is limited with high inter- and intrascanner variabilities, which may even result in reclassification [6–8]. Rutten et al, for example, demonstrated that small variations of the scan position significantly impact the variability of calcium scores with a potential risk reclassification in 9% of the individuals [7].

For energy-integrating detector (EID)-CT, a new, multivendor validated CAC scoring protocol has already been proposed: In a phantom study, van Praagh et al [9] systematically analyzed score reproducibility using different acquisition and reconstruction settings for CAC quantification on EID-CT systems from four major vendors. By using a protocol at a reduced tube voltage (100 kVp), 75% radiation dose, and thin-sliced reconstructions with a higher iterative reconstruction level, they obtained lower intra- and interscanner variability (by 36% and 34%, respectively) as well as improved detectability of calcifications compared to the current standard EID-CT protocol.

Recently, the introduction of photon-counting detector (PCD)-CTs has ushered in a new clinically available scanner generation, offering several advantages compared to previous EID-CT systems, including improved spatial resolution without compromising dose efficiency [10–13], as well as low-dose imaging [14, 15] with better Hounsfield unit (HU) stability, less image noise, and higher contrast-to-noise ratio compared to EID-CT [16–19]. Furthermore, it has already been demonstrated that PCD-CT quantifies coronary calcifications more accurately than EID-CT [20]. Therefore, PCD-CT offers the potential to also further improve quantitative CT assessment, including CAC scoring. In contrast to the previously proposed EID-CT protocol, to the best of our knowledge, there are no new protocol recommendations for PCD-CT-based CAC scoring.

Therefore, this study aimed to propose a radiation dose-reduced protocol with improved reproducibility of scores and to compare these results with corresponding multivendor values from four state-of-the-art EID-CT systems.

## Materials and methods

In this prospective phantom study, no humans or animal subjects were involved. Therefore, institutional review board approval and informed consent were not required.

To ensure comparability, the overall design of the phantom experiments was based on the study conducted by van Praagh et al [9]. The phantom used in this study and all acquisition and reconstruction parameters chosen were identical to those used in the study by van Praagh et al [9].

### Phantom

In this prospective phantom study, a calcium scoring insert was placed in an anthropomorphic chest phantom (QRM). This insert contains nine cylindrical calcifications with varying diameters (5, 3, and 1 mm). Each size appears three times at varying calcium hydroxyapatite (CaHA) densities (800, 400, and 200 mg/cm^3^), as well as two calibration rods, one with a CaHA density of 200 mg/cm^3^ and one with water-equivalent density. All these inserts are placed in a soft-tissue-equivalent material. With an anterior-posterior × lateral diameter of 30 × 20 cm, this chest phantom simulates a small patient. Additionally, medium- and large-sized chest diameters of 35 × 25 cm and 40 × 30 cm, respectively, were simulated by using two extension rings.

### Data acquisition and image reconstruction

The phantoms were scanned on a first-generation, clinical PCD-CT (NAEOTOM Alpha; Siemens Healthineers) with a simulated electrocardiogram (60 beats per minute; electrocardiogram triggering: single phase of 75%) and gantry rotation time of 0.25 s. Since calcium quantification can vary between several scans [7], and to assess interscan variability, each phantom size was scanned five times per protocol. Between scans, a small repositioning of the phantom was applied (approximately 5 mm translational and 2° rotational) to mimic realistic variations in phantom positioning, following previously published methodology [9, 21]. To ensure comparability, the overall design of the phantom experiments, including the repositioning approach, was based on the study conducted by van Praagh et al [9]. Scans were acquired at two different tube voltages (120 kVp and 90 kVp) and four different radiation dose levels. According to van Praagh et al [9], the standard radiation dose was based on clinical protocols: Volumetric CT dose indices (CTDI_Vol_) of 1.5, 3.3, and 7.0 mGy served as 100% radiation dose for the small, medium and large phantoms, respectively. Gradual reduction of CTDI_Vol_ using tube current modulation resulted in the following investigated radiation dose levels: 100%, 75%, 50%, and 25%.

A proprietary offline raw data reconstruction platform (ReconCT version 15.0.57554.0; Siemens Healthineers) was used for all reconstructions. The convolution kernel was set to Qr36 and the virtual monoenergetic image level to 70 keV, which is the current standard reconstruction for PCD-based CAC quantification according to the vendor’s recommendation. All images were reconstructed at different slice thicknesses/increments (3.0/3.0 mm and 1.0/1.0 mm) and different quantum iterative reconstruction (IR) strength levels (1 to 4).

### Image and statistical analysis

CAC quantification was performed using a validated, fully automated quantification method, as previously described [9, 22]. Based on the calcifications’ location and using a standard threshold for CAC scoring of 130 HU [23], the following parameters were reported: Agatston scores, calcium volume (mm^3^) and calcium mass (mg) of each calcification, as well as image noise (standard deviation of HU values measured in a 1.5 cm^2^ region of interest within the water-equivalent insert). Further CAC scoring analysis mainly focused on Agatston scores. Image noise was evaluated using a lower noise target (20 HU for small/medium, 23 HU for the large phantom) to prevent unreasonably high radiation doses [5], and an upper threshold (30 HU for small/medium, 35 HU for the large phantom) to prevent false-positive lesions due to high image noise [9].

GraphPad Prism (Version 8.4.2; GraphPad) was used for statistical analysis. Continuous variables are reported as median with interquartile range (IQR), categorical variables as absolute frequencies and proportions. Using median with IQR down-weights outliers and may understate the impact of individual extremes. Nevertheless, figures additionally illustrate the absolute range (min – max). *p* < 0.05 was considered statistically significant.

The IQR of total Agatston scores from all five interscan variability examinations and all phantom sizes was calculated for each acquisition and reconstruction setting. Scores from different PCD scan protocols were compared to those derived by the PCD-CT standard protocol (120 kVp, 100% dose, 3 mm slice thickness) as well as to corresponding values derived by scanning the same phantoms on state-of-the-art EID-CT scanners from four different vendors (SOMATOM Force, Siemens Healthineers; Revolution, GE Healthcare; iCT, Philips Healthcare; Aquilion One PRISM Edition, Canon Medical Systems) using the standard, as well as the new EID-CT protocol proposed by van Praagh et al [9].

Reproducibility of Agatston scores was investigated using the change of variability according to the following formula [9]:

$$\left(-1+\frac{{{{{{\rm{IQR}}}}}}_{{{{{\rm{new}}}}}\; {{{{\rm{protocol}}}}}}}{{{{{{\rm{IQR}}}}}}_{{{{{\rm{EID}}}}}\; {{{{\rm{standard}}}}}\; {{{{\rm{protocol}}}}}}}\right){{{{\rm{x}}}}}100 \%$$

Furthermore, the number of detected calcified lesions (maximum of nine per phantom) and the image noise values were used for further comparison of different protocols. The number of detected calcifications between different protocols was compared using a paired *t*-test and between different dose and IR levels using one-way ANOVA with Geisser-Greenhouse correction. Mann–Whitney U test was used to analyze differences in image noise and Agatston scores between different protocols.

## Results

### Reproducibility of CAC quantification

**Fig. 1:**
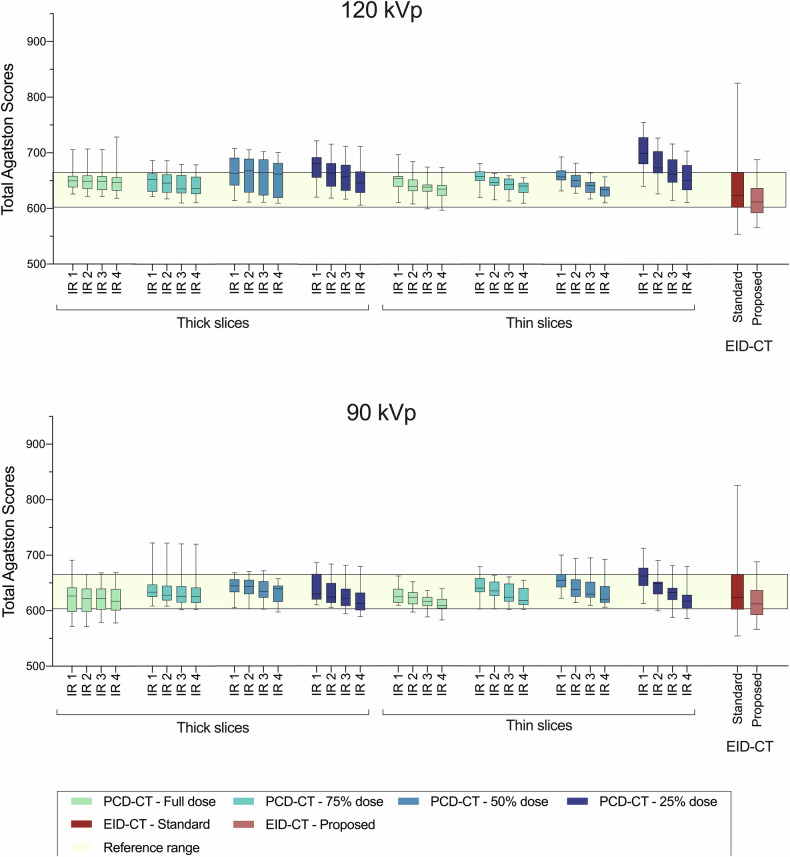
Total Agatston scores of all phantom sizes scanned with photon-counting detector (PCD)-CT protocols at different tube voltages (120 kVp, 90 kVp), slice thicknesses (thick: 3.0 mm, thin: 1.0 mm), radiation doses (100%, 75%, 50%, 25%) and iterative reconstruction (IR) levels (strength 1 to 4) in comparison to Agatston scores obtained from four state-of-the-art energy-integrating detector (EID)-CT scanners by using the standard and previously proposed EID-CT protocol. The light-yellow range represents the interquartile range (IQR) of all Agatston scores from those four EID-CT scanners using the standard EID-CT protocol. IR, iterative reconstruction

Figure 1 illustrates Agatston score variability at different acquisition and reconstruction settings in comparison with the IQR acquired by using the standard and proposed EID-CT protocol.

Solely reducing the tube voltage resulted in an increase of Agatston score variability by 67% (61% to 82%) compared to the PCD-CT standard protocol. Decreasing slice thickness lowered the score variability by −18% (−27% to −14%) for the 120 kVp protocol and by −52% (−56% to −47%) for the 90 kVp protocol, each compared to the corresponding thick-sliced protocol. Compared to the 90 kVp thin-sliced protocol, the 120 kVp thin-sliced protocol shows a lower score variability by −15% (−26% to 0%). Therefore, the manuscript will primarily focus on the analyses at 120 kVp.

Comparing this 120 kVp, thin-slice, 100% dose protocol with reduced radiation dose levels of 75%, 50%, and 25%, resulted in Agatston score variability changes of −8% (−14% to 11%), 0% (−7% to 18%), and 150% (128% to 182%), respectively. Similar observations were observed in calcium volumes and masses (see Supplementary Results).

Per IR level, using a 120 kVp, thin-sliced protocol at 75% at IR 1, IR 2, IR 3, and IR 4, resulted in Agatston score variability changes of −10%, −26%, 64%, and −6%, respectively, compared to respective values acquired using the 120 kVp, thin-sliced protocol at 100% dose.

### Image noise

**Fig. 2:**
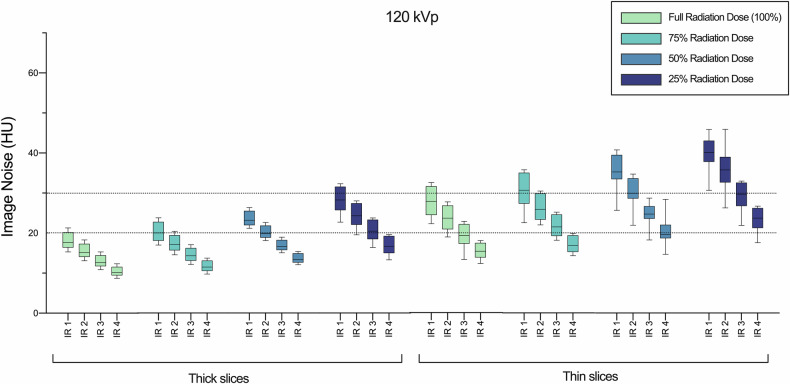
Image noise levels of all phantom sizes scanned with protocols at 120 kVp and different slice thicknesses (thick: 3.0 mm; thin: 1.0 mm), radiation doses (100%, 75%, 50%, 25%) and iterative reconstruction (IR) levels (strength 1 to 4). The two dashed lines show the target range

Image noise was below the upper threshold with standard radiation dose and thick slices at 120 kVp (Fig. 2). Protocols that exceeded the upper threshold, indicating a higher risk of false-positives, were excluded from further protocol recommendations. At reduced radiation doses, thin-sliced protocols at 120 kVp exceeded the upper noise threshold with IR strength 1. When using IR strength 2, thin-sliced protocols at 120 kVp with 50% and 25% dose resulted in a median image noise exceeding the upper threshold. Only thin-sliced protocols at 120 kVp with 75% dose stayed within the noise thresholds when using IR 2.

### Calcification detectability

The number of detected calcifications per protocol is shown in Table 1. Using the PCD-CT standard protocol 6.0 ± 0.0 of the nine calcifications were detected. Solely reducing the slice thickness resulted in an increase of detected calcifications on average by 0.8 ± 0.7 for the 120 kVp protocol (*p* < 0.001). A significant increase in detectability was found at reduced radiation doses, when looking at the 120 kVp, thin slices protocol using IR 1 or 2 (IR1: *p* = 0.007, IR2: *p* = 0.003). However, no significant differences were found for the same comparison with IR 3 or 4 (IR3: *p* = 0.75, IR4: *p* = 0.51). The detectability of calcifications decreased significantly with rising IR level (*p* < 0.001) and was best for IR ≤ 2.

**Table 1:** Number of detected calcifications per protocol at 120 kVp

kVp	Dose	Reconstruction	Number of calcifications		
			3.0 mm		1.0 mm
120	100%	IR 1	6.0 ± 0.0		7.3 ± 0.5
		IR 2	6.0 ± 0.0		7.2 ± 0.6
		IR 3	6.0 ± 0.0		6.5 ± 0.6
		IR 4	6.0 ± 0.0		6.3 ± 0.5
	75%	IR 1	6.0 ± 0.0		7.4 ± 0.7
		IR 2	6.0 ± 0.0		7.1 ± 0.8
		IR 3	6.0 ± 0.0		6.4 ± 0.5
		IR 4	6.0 ± 0.0		6.1 ± 0.4
	50%	IR 1	6.0 ± 0.4		7.7 ± 0.8
		IR 2	6.0 ± 0.0		6.7 ± 0.6
		IR 3	6.0 ± 0.0		6.5 ± 0.5
		IR 4	5.9 ± 0.3		6.1 ± 0.3
	25%	IR 1	6.2 ± 0.4		8.1 ± 0.6
		IR 2	6.0 ± 0.4		7.7 ± 0.7
		IR 3	6.0 ± 0.4		6.6 ± 0.6
		IR 4	5.9 ± 0.3		6.1 ± 0.4

Numbers are presented as mean ± standard deviation of repeated scans and phantom size

*IR* iterative reconstruction

### Optimized protocol—comparison to the PCD-CT standard protocol as well as EID-CT protocols

**Fig. 3:**
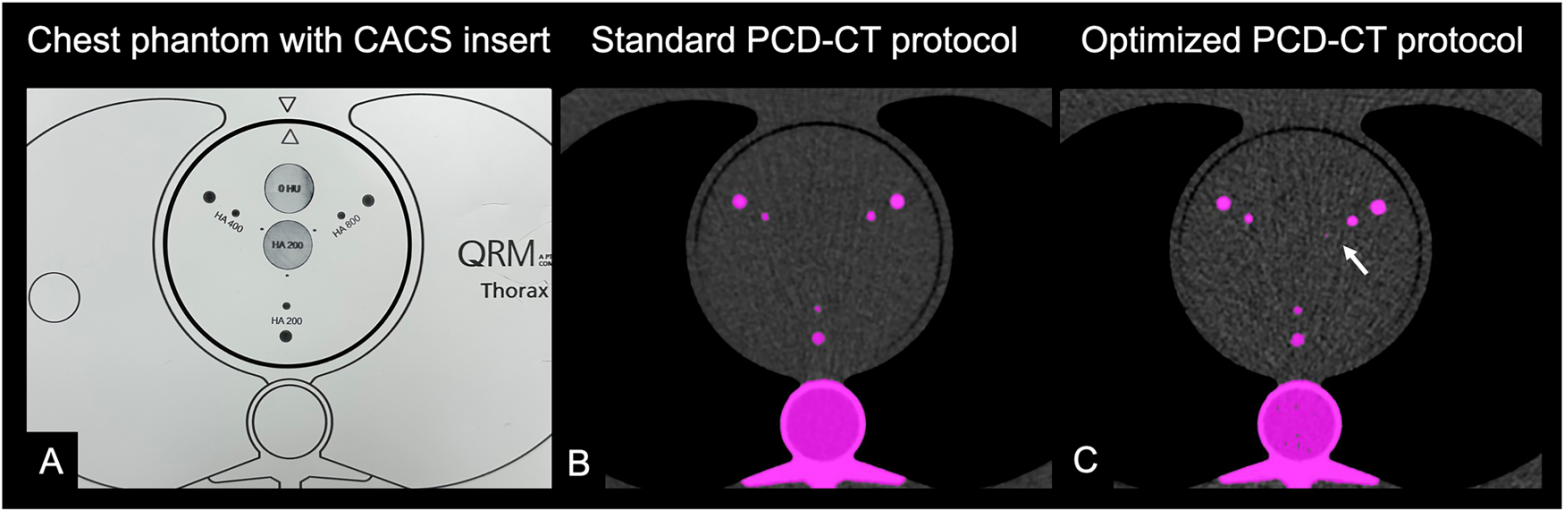
Picture of the chest phantom including the calcium scoring insert (**A**) and axial reconstructions illustrating sample scans acquired using the standard (**B**) and the optimized photon-counting detector (PCD)-CT protocol (**C**), demonstrating improved calcification detectability (arrow). CAC, coronary artery calcium; QRM, anthropomorphic chest phantom

Based on the results mentioned above, settings at 120 kVp, thin slices (1.0 mm), 75% dose, and IR strength 2 were chosen as the new recommended PCD-CT protocol. Figure 3 illustrates axial reconstructions of each phantom size with the standard and the optimized protocol.

This optimized PCD-CT protocol resulted in an improved Agatston score variability by 37% compared to the standard PCD-CT protocol, by 76% compared to the standard EID-CT protocol, and by 66% compared to the previously proposed EID-CT protocol, each of all four scanners. When comparing the optimized PCD-CT protocol with the most comparable EID-CT scanner from the same vendor (SOMATOM Force, Siemens Healthineers), the results demonstrated a 26% reduction in Agatston score variability compared to the EID-CT standard protocol and a 16% reduction compared to the previously proposed EID-CT protocol. Accordingly, improvements in the variability of calcium volumes and masses were also found (see Supplementary Results).

Using the optimized PCD-CT protocol, image noise was 23.3 (22.7–23.8), 26.0 (24.8–26.5), and 30.2 HU (29.3–30.4) for the small-, medium-, and large-sized phantom, respectively. Compared to noise levels from scans obtained with the proposed EID-CT protocol, image noise using the optimized PCD-CT protocol was similar for the small and medium phantom (*p* = 0.69 and *p* = 0.91) but significantly higher for the large phantom (30.2 HU [29.3–30.4] vs. 28.4 HU [27.0–28.8]; *p* = 0.008). However, even the higher image noise was below the upper noise threshold for the large phantom [9].

The number of detected calcifications was 7.1 ± 0.8 using the optimized PCD-CT protocol, 6.0 ± 0.0 using the standard PCD-CT protocol, 6.2 ± 0.4 using the standard EID-CT protocol and 7.0 ± 0.4 using the proposed EID-CT protocol.

### Per-calcification analysis

Using the optimized PCD-CT protocol, median Agatston scores of 800 mg/cm^3^ CaHA decreased by 9% and 11% for large- and medium-sized calcifications, respectively, compared to the PCD-CT standard protocol. Respective median Agatston scores of 400 mg/cm^3^ CaHA increased by 3% and 19% for large- and medium-sized calcifications, respectively. Median Agatston scores of 200 mg/cm^3^ CaHA increased by 55% and 28% for large- and medium-sized calcifications, respectively. Overall median Agatston scores did not significantly differ between the standard and the optimized PCD-CT protocol (*p* = 0.93).

Calcification volumes and mass scores of the optimized PCD-CT protocol were closer to the physical volume and mass of all calcifications (see Supplementary Results and Supplementary Figs. S1 and S2).

**Fig. 4:**
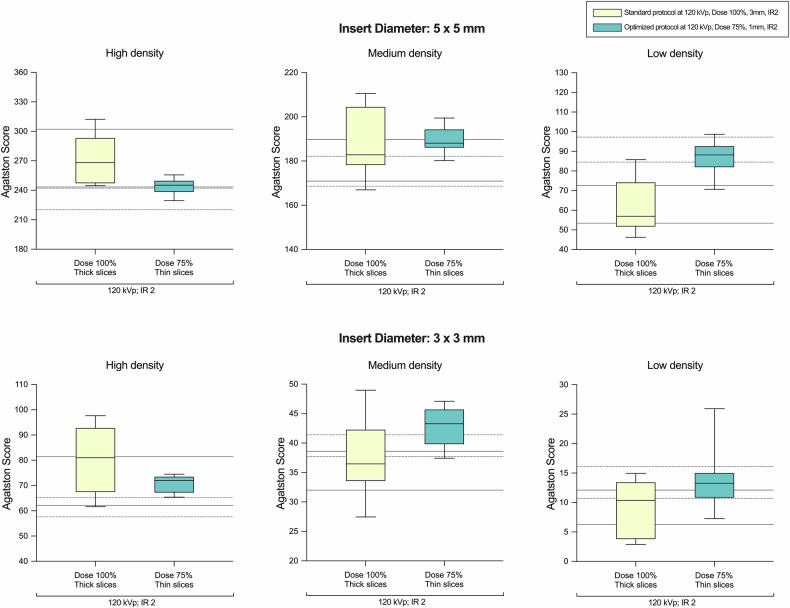
Per calcification analysis of Agatston score. The continuous lines illustrate the interquartile range of the Agatston scores using the energy-integrating detector (EID-CT) standard protocol. The dashed lines show the interquartile range of the Agatston scores using the previously proposed EID-CT protocol. The illustration of the 1 mm diameter calcifications was omitted, as these calcifications have not been detectable with every protocol. Calcifications with 800 mg/m³ calcium hydroxyapatite (CaHA) are defined as “High density,” with 400 mg/m³ CaHA as “Medium density” and with 200 mg/m³ CaHA as “Low density”. IR, iterative reconstruction

Compared to the standard PCD-CT protocol, Agatston score variability decreased by 74%, 64%, and 55% for large-sized 800, 400, and 200 mg/cm^3^ CaHA, and by 80%, 26%, and 61% for medium-sized 800, 400, and 200 mg/cm^3^ CaHA calcifications, respectively. Corresponding changes compared to the standard EID-CT protocol were −83%, −62%, −52% and −75%, −32%, −43%, and compared to the proposed EID-CT protocol −54%, −47%, −28% and −35%, 32%, −61%. The comparison of the Agatston score variabilities between the different protocols is illustrated in Fig. 4.

## Discussion

CAC scoring is widely used for cardiovascular risk assessment but has limited reproducibility. This study provides a dose-reduced protocol with improved CAC score variability, outperforming previous PCD- and EID-CT protocols. The main findings are: First, the lowest score variability was obtained using a 120 kVp, thin-sliced PCD-CT protocol at 75% dose. Second, image noise thresholds preventing false-positives were not exceeded at IR ≥ 2. Third, the detectability of calcifications was highest at IR ≤ 2. Fourth, based on these results, a protocol at 120 kVp, thin slices, 75% dose, and IR2 was proposed, resulting in an improved reproducibility by 37% compared to the standard PCD-CT protocol, by 76% compared to the standard EID-CT protocol, and by 66% compared to the previously proposed EID-CT protocol, together with a higher calcification detectability. Fifth, volume and mass scores of the optimized PCD-CT protocol were closer to the physical reference.

CAC scoring guides clinical decision-making [4]. Precise and robust CAC scoring is essential for optimal patient care. However, the current scoring standard has not been updated for years, although limited reproducibility may lead to risk reclassification [7, 8], possibly resulting in different treatment decisions. These aspects highlight the need for a new standard in CAC scoring [6, 24]. To our knowledge, the present study is the first to expand recommendations regarding a new CAC scoring method for PCD-CT.

In this study, score variability increased with lower tube voltages but improved with decreased slice thickness. This is in line with previous EID-CT studies. In a phantom study [9], intrascanner variability increased by 7% after reducing the tube voltage but decreased by 54% when additionally reducing slice thickness. Vonder et al [25] observed a slight increase in variability when reducing the tube voltage from 120 to 90 and 100 kVp, respectively (4.0% vs. 7.8% vs. 5.4%). Other studies, which observed an impact on cardiovascular risk reclassification in some cases when reducing the tube voltage in CAC scoring [26, 27], support this hypothesis. In our study, the difference in score variability using a protocol reduced to 90 kVp compared to 120 kVp, with an increase of 67%, was even more pronounced, indicating that this effect might be more significant with PCD-CT. Kim et al already showed that CAC scoring using thin-sliced reconstructions is feasible [28, 29]. Despite several studies demonstrating decreasing score variability with reduced slice thickness [9, 30, 31], this was not sufficient to counteract the significant difference between 90 and 120 kVp in our study. Therefore, we recommend maintaining the use of 120 kVp.

Radiation dose reduction to 75% additionally decreased score variability, which is particularly relevant given the remaining concerns regarding radiation dose exposure despite the proven clinical value of CAC scoring. Further decreasing the dose without exceeding noise thresholds, indicating an increased risk of false-positives, was only feasible with IR ≥ 2. However, calcification detectability decreases with increasing iterative reconstruction levels, which has been demonstrated in our analysis and previous studies [9, 25]. In addition, Urabe et al already demonstrated that lowering the slice thickness improves the detection of small calcifications [32]. In the present study, detectability especially improved for small and low-density calcifications when using the optimized PCD-CT protocol. Furthermore, the per-calcification analysis in our study showed decreasing Agatston scores for high-density plaques and increasing scores for low-density plaques. Together with the closer agreement of volume and mass scores to the physical reference, this indicates a bias reduction and improved detection of subtle plaques. As the absence of CAC is associated with a very low risk [33–36] and may lead to fewer recommendations regarding preventive therapies [37, 38], the distinction between the absence and low CAC is essential, as patients with a CAC score between one and ten have been shown to have higher event rates than those with a CAC score of zero [33, 35, 39–41]. Furthermore, the individual cardiovascular risk is higher in the presence of low-density plaques [42].

Overall, the protocol recommended by this study resulted in an improved score variability and calcification detectability compared to current PCD-CT and EID-CT protocols, without exceeding the image noise thresholds. Furthermore, volume and mass scores from the optimized protocol were closer to the physical reference, resulting in a more precise presentation of calcifications.

While the optimized PCD-CT protocol demonstrates technical advantages of PCD-CT for reproducible CAC scoring, its widespread implementation in clinical routine depends on scanner availability and capacities to perform CAC scoring studies to further clinically validate these results. In most centers, CAC scoring will most likely still be performed using EID-CT scanners, which underlines the importance of ongoing optimization across both detector technologies. Even though its clinical implementation may take time, the optimized PCD-CT protocol represents a logical further development of existing EID-CT-based recommendations.

Our study has several limitations. A static phantom with specific calcifications was used. The impact of cardiac motion was not investigated. Future studies should investigate these aspects. We did not investigate the optimized PCD-CT protocol in patients. However, this in vitro analysis provides first systematic results and represents an essential basis for further in vivo studies. The settings in this study were specifically chosen to ensure comparability to the study by van Praagh et al [9] investigating a new CAC scoring protocol for EID-CT. This includes matching slice thickness and increment, as well as manually selecting radiation doses. However, these settings may not fully reflect clinical practice, where automatic exposure control systems are routinely applied. Additionally, oversampling techniques, such as using a lower increment than slice thickness, could further reduce score variability. In addition, this study was performed on a single-vendor, first-generation PCD-CT system. Scanner-specific differences, such as temporal resolution [43], may influence CAC quantification. Therefore, generalizability to other PCD-CT systems needs to be validated in further studies. To ensure clinical applicability, future studies should investigate the optimized PCD-CT protocol under real-world clinical conditions.

In conclusion, using a 120 kVp protocol with thin slices, 75% dose, and IR2 for PCD-CT CAC scoring was associated with lower score variability and higher calcification detectability compared to current PCD-CT and EID-CT protocols. While further validation and broader availability of PCD-CT will be necessary, these results represent a meaningful step toward a more precise and consistent CAC quantification.

## Supplementary information

ELECTRONIC SUPPLEMENTARY MATERIAL

## Abbreviations

CAC: Coronary artery calcium
CaHA: Calcium hydroxyapatite
CTDI_Vol_: Volumetric CT dose index
EID: Energy-integrating detector
HU: Hounsfield unit
IQR: Interquartile range
IR: Iterative reconstruction
PCD: Photon-counting detector

## References

[1] Osborne-Grinter M, Ali A, Williams MC (2024) Prevalence and clinical implications of coronary artery calcium scoring on non-gated thoracic computed tomography: a systematic review and meta-analysis. Eur Radiol 34:4459–4474 38133672 10.1007/s00330-023-10439-z PMC11213779

[2] Tummala R, Han D, Friedman J et al (2022) Association between plaque localization in proximal coronary segments and MACE outcomes in patients with mild CAC: results from the EISNER study. Am J Prev Cardiol 12:100423 36199447 10.1016/j.ajpc.2022.100423 PMC9529495

[3] Budoff MJ, Young R, Burke G et al (2018) Ten-year association of coronary artery calcium with atherosclerotic cardiovascular disease (ASCVD) events: the multi-ethnic study of atherosclerosis (MESA). Eur Heart J 39:2401–2408 29688297 10.1093/eurheartj/ehy217 PMC6030975

[4] Arnett DK, Blumenthal RS, Albert MA et al (2019) 2019 ACC/AHA guideline on the primary prevention of cardiovascular disease. J Am Coll Cardiol 74:e177–e232 30894318 10.1016/j.jacc.2019.03.010 PMC7685565

[5] McCollough CH, Ulzheimer S, Halliburton SS, Shanneik K, White RD, Kalender WA (2007) Coronary artery calcium: a multi-institutional, multimanufacturer international standard for quantification at cardiac CT. Radiology 243:527–538 10.1148/radiol.2432050808 17456875

[6] Willemink MJ, van der Werf NR, Nieman K, Greuter MJW, Koweek LM, Fleischmann D (2019) Coronary artery calcium: a technical argument for a new scoring method. J Cardiovasc Comput Tomogr 13:347–352 10.1016/j.jcct.2018.10.014 30366859

[7] Rutten A, Isgum I, Prokop M (2008) Coronary calcification: effect of small variation of scan starting position on Agatston, volume, and mass scores. Radiology 246:90–98 18024437 10.1148/radiol.2461070006

[8] Willemink MJ, Vliegenthart R, Takx RAP et al (2014) Coronary artery calcification scoring with state-of-the-art CT scanners from different vendors has substantial effect on risk classification. Radiology 273:695–702 25153157 10.1148/radiol.14140066

[9] van Praagh GD, Wang J, van der Werf NR et al (2022) Coronary artery calcium scoring: toward a new standard. Invest Radiol 57:13–22 10.1097/RLI.0000000000000808 PMC10072789 34261083

[10] Rajendran K, Petersilka M, Henning A et al (2022) First clinical photon-counting detector CT system: technical evaluation. Radiology 303:130–138 34904876 10.1148/radiol.212579 PMC8940675

[11] Willemink MJ, Persson M, Pourmorteza A, Pelc NJ, Fleischmann D (2018) Photon-counting CT: technical principles and clinical prospects. Radiology 289:293–312 10.1148/radiol.2018172656 30179101

[12] Wang J, Huang Z, Zhu Z et al (2025) Photon-counting detector CT provides superior subsolid nodule characterization compared to same-day energy-integrating detector CT. Eur Radiol 35:2979–2989 39609282 10.1007/s00330-024-11204-6

[13] Chang S, Ren L, Tang S et al (2023) Technical note: Exploring the detectability of coronary calcification using ultra-high-resolution photon-counting-detector CT. Med Phys 50:6836–6843 37650788 10.1002/mp.16712 PMC10841095

[14] Symons R, Sandfort V, Mallek M, Ulzheimer S, Pourmorteza A (2019) Coronary artery calcium scoring with photon-counting CT: first in vivo human experience. Int J Cardiovasc Imaging 35:733–739 10.1007/s10554-018-1499-6 30635819

[15] Hellms S, Werncke T, Böttcher J et al (2026) Reduction of radiation exposure and preserved image quality using photon-counting detector cardiac computed tomography without electrocardiographic gating in children with congenital heart disease. Eur Radiol 36:324–333 10.1007/s00330-025-11719-6 PMC12712116 40608094

[16] Symons R, Cork TE, Sahbaee P et al (2016) Low-dose lung cancer screening with photon-counting CT: a feasibility study. Phys Med Biol 62:202 27991453 10.1088/1361-6560/62/1/202 PMC5237389

[17] Symons R, Pourmorteza A, Sandfort V et al (2017) Feasibility of dose-reduced chest CT with photon-counting detectors: initial results in humans. Radiology 285:980–989 28753389 10.1148/radiol.2017162587 PMC5708286

[18] McCollough CH, Rajendran K, Baffour FI et al (2023) Clinical applications of photon counting detector CT. Eur Radiol 33:5309–5320 37020069 10.1007/s00330-023-09596-y PMC10330165

[19] McCollough CH, Rajendran K, Leng S et al (2023) The technical development of photon-counting detector CT. Eur Radiol 33:5321–5330 37014409 10.1007/s00330-023-09545-9 PMC10330290

[20] Sandstedt M, Marsh J, Rajendran K et al (2021) Improved coronary calcification quantification using photon-counting-detector CT: an ex vivo study in cadaveric specimens. Eur Radiol 31:6621–6630 33713174 10.1007/s00330-021-07780-6 PMC8380662

[21] Groen J, Greuter M, Vliegenthart R et al (2008) Calcium scoring using 64-slice MDCT, dual source CT and EBT: a comparative phantom study. Int J Cardiovasc Imaging 24:547–556 18038190 10.1007/s10554-007-9282-0 PMC2373860

[22] van Praagh GD, van der Werf NR, Wang J et al (2021) Fully automated quantification method (FQM) of coronary calcium in an anthropomorphic phantom. Med Phys 48:3730–3740 33932026 10.1002/mp.14912 PMC8360117

[23] Agatston AS, Janowitz WR, Hildner FJ, Zusmer NR, Viamonte Jr M, Detrano R (1990) Quantification of coronary artery calcium using ultrafast computed tomography. J Am Coll Cardiol 15:827–832 10.1016/0735-1097(90)90282-t 2407762

[24] Blaha MJ, Mortensen MB, Kianoush S, Tota-Maharaj R, Cainzos-Achirica M (2017) Coronary artery calcium scoring: is it time for a change in methodology? JACC Cardiovasc Imaging 10:923–937 10.1016/j.jcmg.2017.05.007 28797416

[25] Vonder M, Pelgrim GJ, Meyer M et al (2017) Dose reduction techniques in coronary calcium scoring: the effect of iterative reconstruction combined with low tube voltage on calcium scores in a thoracic phantom. Eur J Radiol 93:229–235 28668419 10.1016/j.ejrad.2017.06.001

[26] Marwan M, Mettin C, Pflederer T et al (2013) Very low-dose coronary artery calcium scanning with high-pitch spiral acquisition mode: comparison between 120-kV and 100-kV tube voltage protocols. J Cardiovasc Comput Tomogr 7:32–38 23333186 10.1016/j.jcct.2012.11.004

[27] Gräni C, Vontobel J, Benz DC et al (2018) Ultra-low-dose coronary artery calcium scoring using novel scoring thresholds for low tube voltage protocols—a pilot study. Eur Heart J Cardiovasc Imaging 19:1362–1371 29432592 10.1093/ehjci/jey019

[28] Choi H, Park EA, Ahn C et al (2023) Performance of 1-mm non-gated low-dose chest computed tomography using deep learning-based noise reduction for coronary artery calcium scoring. Eur Radiol 33:3839–3847 36520181 10.1007/s00330-022-09300-6

[29] Kim C, Hong S, Choi H et al (2025) Impact of deep learning-based image conversion on fully automated coronary artery calcium scoring using thin-slice, sharp-kernel, non-gated, low-dose chest CT scans: a multi-center study. Korean J Radiol 26:759–770 40527737 10.3348/kjr.2025.0177 PMC12318652

[30] Aslam A, Khokhar US, Chaudhry A et al (2014) Assessment of isotropic calcium using 0.5-mm reconstructions from 320-row CT data sets identifies more patients with non-zero Agatston score and more subclinical atherosclerosis than standard 3.0-mm coronary artery calcium scan and CT angiography. J Cardiovasc Comput Tomogr 8:58–66 24582044 10.1016/j.jcct.2013.12.007

[31] Horiguchi J, Matsuura N, Yamamoto H et al (2008) Variability of repeated coronary artery calcium measurements by 1.25-mm- and 2.5-mm-thickness images on prospective electrocardiograph-triggered 64-slice CT. Eur Radiol 18:209–216 17674003 10.1007/s00330-007-0734-7

[32] Urabe Y, Yamamoto H, Kitagawa T et al (2016) Identifying small coronary calcification in non-contrast 0.5-mm slice reconstruction to diagnose coronary artery disease in patients with a conventional zero coronary artery calcium score. J Atheroscler Thromb 23:1324–1333 27397477 10.5551/jat.35808 PMC5221495

[33] Blaha MJ, Cainzos-Achirica M, Dardari Z et al (2020) All-cause and cause-specific mortality in individuals with zero and minimal coronary artery calcium: a long-term, competing risk analysis in the Coronary Artery Calcium Consortium. Atherosclerosis 294:72–79 31784032 10.1016/j.atherosclerosis.2019.11.008 PMC7012761

[34] Blaha MJ, Cainzos-Achirica M, Greenland P et al (2016) Role of coronary artery calcium score of zero and other negative risk markers for cardiovascular disease. Circulation 133:849–858 26801055 10.1161/CIRCULATIONAHA.115.018524 PMC4775391

[35] Joshi PH, Blaha MJ, Budoff MJ et al (2017) The 10-year prognostic value of zero and minimal CAC. JACC Cardiovasc Imaging 10:957–958 28797418 10.1016/j.jcmg.2017.04.016

[36] Valenti V, ó Hartaigh B, Heo R et al (2015) A 15-year warranty period for asymptomatic individuals without coronary artery calcium: a prospective follow-up of 9,715 individuals. JACC Cardiovasc Imaging 8:900–909 26189116 10.1016/j.jcmg.2015.01.025 PMC4537357

[37] Nasir K, Bittencourt MS, Blaha MJ et al (2015) Implications of coronary artery calcium testing among statin candidates according to American College of Cardiology/American Heart Association cholesterol management guidelines: MESA (Multi-Ethnic Study of Atherosclerosis). J Am Coll Cardiol 66:1657–1668 26449135 10.1016/j.jacc.2015.07.066

[38] Cainzos-Achirica M, Miedema MD, McEvoy JW et al (2020) Coronary artery calcium for personalized allocation of aspirin in primary prevention of cardiovascular disease in 2019. Circulation 141:1541–1553 32233663 10.1161/CIRCULATIONAHA.119.045010 PMC7217722

[39] Blaha M, Budoff MJ, Shaw LJ et al (2009) Absence of coronary artery calcification and all-cause mortality. JACC Cardiovasc Imaging 2:692–700 19520338 10.1016/j.jcmg.2009.03.009

[40] Budoff MJ, McClelland RL, Nasir K et al (2009) Cardiovascular events with absent or minimal coronary calcification: the Multi-Ethnic Study of Atherosclerosis (MESA). Am Heart J 158:554–561 19781414 10.1016/j.ahj.2009.08.007 PMC2766514

[41] Han D, Klein E, Friedman J et al (2020) Prognostic significance of subtle coronary calcification in patients with zero coronary artery calcium score: from the CONFIRM registry. Atherosclerosis 309:33–38 32862086 10.1016/j.atherosclerosis.2020.07.011

[42] Razavi AC, van Assen M, De Cecco CN et al (2022) Discordance between coronary artery calcium area and density predicts long-term atherosclerotic cardiovascular disease risk. JACC Cardiovasc Imaging 15:1929–1940 35850937 10.1016/j.jcmg.2022.06.007 PMC9883836

[43] Sartoretti T, Mergen V, Dzaferi A et al (2025) Effect of temporal resolution on calcium scoring: insights from photon-counting detector CT. Int J Cardiovasc Imaging 41:615–625 10.1007/s10554-024-03070-6 PMC11880162 38389028

