# Supplementary material for: Coronary artery calcium scoring: expanding the new standard by photon-counting detector CT—Part I: Impact of tube voltage, tube current, slice thickness, and quantum iterative reconstructions
Source: Eur Radiol. 2026 Feb 19;36(7):5546–56. doi: 10.1007/s00330-026-12355-4 (PMC13282307; doi:10.1007/s00330-026-12355-4)
Supplement: Supplementary file 1 — ELECTRONIC SUPPLEMENTARY MATERIAL [file 330_2026_12355_MOESM1_ESM.pdf]

Coronary Artery Calcium Scoring: Expanding the New Standard by Photon-Counting Detector CT Part I: Impact of Tube Voltage, Tube Current, Slice Thickness, and Quantum Iterative Reconstructions

ELECTRONIC SUPPLEMENTARY MATERIAL

Supplementary Results

Reproducibility of CAC quantification

Overall, analyses of calcium volumes showed similar trends as total Agatston score analyses. Reducing the tube voltage to 90kVp increased the calcium volume variability by 24% (21% to 25%) compared to the PCD-CT standard protocol. Compared to each thick sliced protocol, lowering the slice thickness resulted in a decrease of volume variability by -33% (-37% to -30%) for the 120kVp protocol and by -27% (-28% to -24%) for the 90kVp protocol. The 90kVp thin sliced protocol showed 33% (31% to 42%) higher volume variability than the 120kVp thin sliced protocol. Comparing this 120kVp, thin slice, 100% dose protocol with reduced radiation doses of 75%, 50%, and 25% resulted in volume score changes of -24% (-27% to -13%), 121% (111% to 131%), and 218% (202% to 246%), respectively.

Corresponding calcium mass variability was as follows. Reducing only the tube voltage resulted in an increasing variability by 121% (119% to 122%). Reducing the slice thickness decreased mass variability by -35% (-39% to -25%) for the 120kVp protocol and by -30% (-34% to -26%) for the 90kVp protocol, each compared to the corresponding thick sliced protocol. The 90kVp thin sliced protocol showed a 138% (115% to 147%) higher mass variability than the corresponding 120kVp protocol. Comparing this 120kVp, thin slice, 100% dose protocol with reduced radiation doses of 75%, 50%, and 25% resulted in volume variability changes of 50% (47% to 61%), 0% (-18% to 14%), and 89% (57% to 119%), respectively.

Optimized protocol

The optimized PCD-CT protocol decreased volume variability by 38% compared to the PCD-CT standard protocol, by 89% compared to the EID-CT standard protocol and by 76% compared to the previously proposed EID-CT protocol of all four state-of-the-art EID-CT scanners.

The optimized protocol resulted in mass variability changes by 31% compared to the PCD-CT standard protocol, by -69% compared to the EID-CT standard protocol and by -65% compared to the previously proposed EID-CT protocol of all four EID-CT scanners.

Per-Calcification Analysis

Median volume scores of 800 mg/cm^3 CaHA decreased by 16% for large- and by 22% for medium-sized calcifications when using the optimized PCD-CT protocol compared to the standard PCD protocol. Corresponding median volumes of 400 mg/cm^3 CaHA decreased by 11% and 1% for large- and medium-sized calcifications, respectively. Median volumes of 200 mg/cm^3 CaHA changed by 6% for large- and by 82% for medium-sized calcifications. Compared to the standard PCD-CT protocol, volume variability changed by -82%, 51%, and -57% for large-sized 800, 400, and 200 mg/cm^3 CaHA, and by -88%, 14%, and -7% for medium-sized 800, 400, and 200 mg/cm^3 CaHA calcifications, respectively. Corresponding changes were -92%, -82%, -84% and -93%, -64%, -5% compared to the standard EID-CT protocol, and -76%, -68%, -76% and -88%, 31%, -10% compared to the proposed EID-CT protocol.

Using the optimized PCD-CT protocol compared to the standard PCD protocol, median mass scores of 800 mg/cm^3 CaHA changed by 0% and 4% for large- and medium-sized calcifications, respectively. Median mass scores of 400 mg/cm^3 CaHA increased by 3% for large-sized and by 11% for medium-sized calcifications. Corresponding median masses of 200 mg/cm^3 CaHA changed by 12% and 40% for large- and medium-sized calcifications, respectively. Compared to the standard PCD protocol, mass score variability changed by -59%, 110%, and -61% for large-sized 800, 400, and 200 mg/cm^3 CaHA, and by -16%, 26%, and -33% for medium-sized 800, 400, and 200 mg/cm^3 CaHA calcifications, respectively. Corresponding changes of mass variability were -75%, -55%, -75% and -61%, -48%, -12% when comparing to the standard EID-CT protocol, and -74%, -36%, -59%, and -64%, 4%, -25% when comparing to the proposed EID-CT protocol.

Supplementary Figures

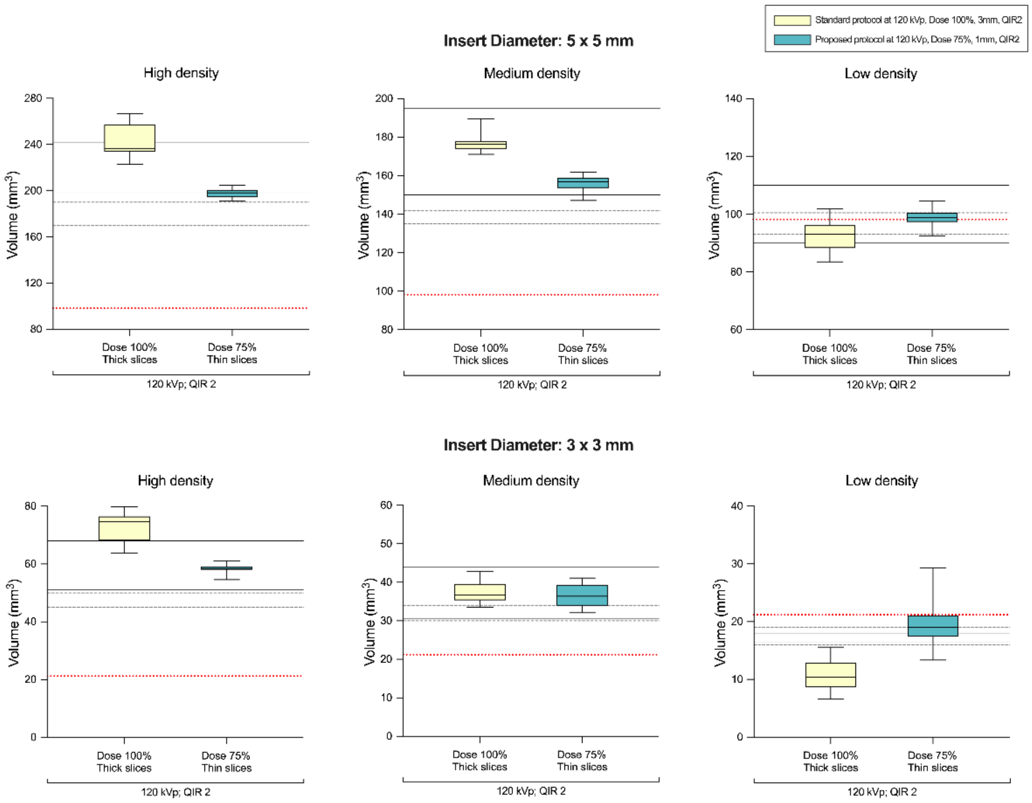

Figure S1: Per calcification analysis of calcium volume scores. The continuous lines illustrate the interquartile range of the volume scores using the energy-integrating detector (EID)-CT standard protocol. The dashed lines show the interquartile range of the volume scores using the previously optimized EID-CT protocol. The red lines illustrate the physical volume of investigated calcifications. The illustration of the 1 mm diameter calcifications was omitted, as these calcifications have not been detectable with every protocol.

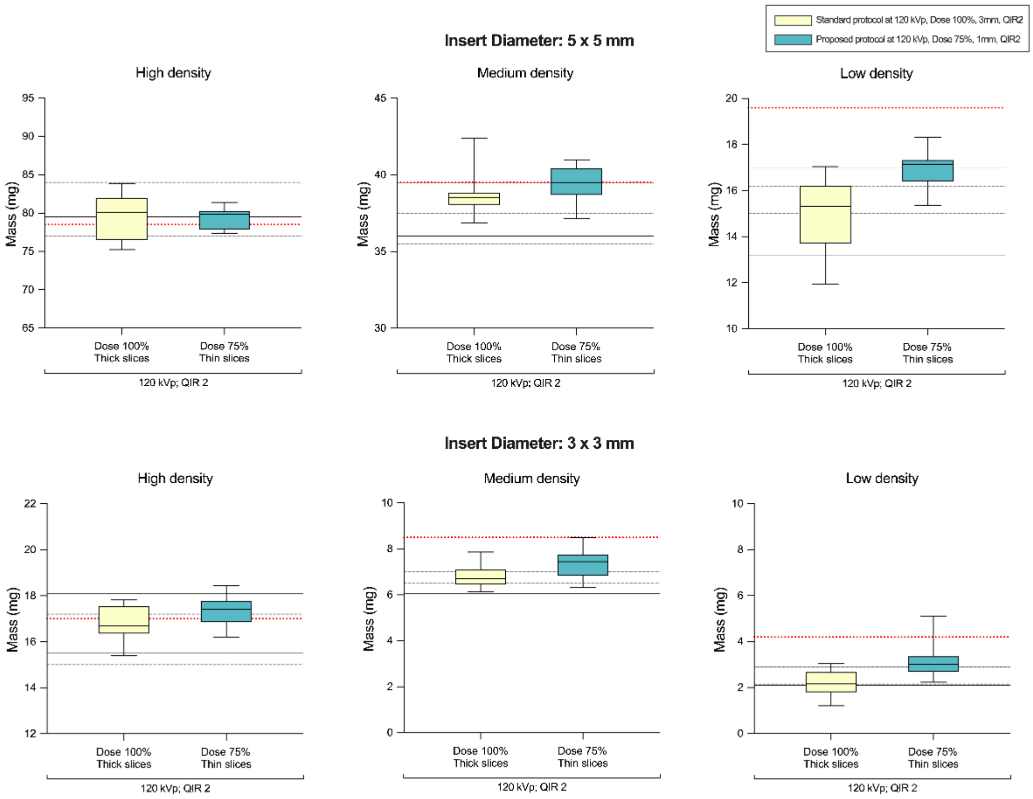

Figure S2: Per calcification analysis of calcium mass scores. The continuous lines illustrate the interquartile range of the mass scores using the energy-integrating detector (EID)-CT standard protocol. The dashed lines show the interquartile range of the mass scores using the previously proposed EID-CT protocol. The red lines illustrate the physical mass of investigated calcifications. The illustration of the 1 mm diameter calcifications was omitted, as these calcifications have not been detectable with every protocol.
